# Front-line therapies for elderly patients with transplant-ineligible multiple myeloma and high-risk cytogenetics in the era of novel agents

**DOI:** 10.1038/s41375-018-0098-9

**Published:** 2018-03-28

**Authors:** Herve Avet-Loiseau, Thierry Facon

**Affiliations:** 1 Unite de Genomique du Myelome, CHU Rangueil, Toulouse, France; 2 Service des Maladies du Sang, Hôpital Claude Huriez, Lille, France

## Abstract

In multiple myeloma, certain cytogenetic abnormalities, such as t(4;14), t(14;16), and del(17p), are considered high risk and are associated with worse prognosis. Patients with these high-risk cytogenetic abnormalities, as well as those who are elderly and transplant ineligible, have not experienced the same degree of improved survival outcomes that other patients have seen with recent advances in the treatment of multiple myeloma. To date, no treatment regimen has demonstrated sustained and consistent survival benefits in elderly, transplant-ineligible patients with high-risk cytogenetic abnormalities and newly diagnosed multiple myeloma. Thus, there is an unmet need to identify effective treatment options for these patients and achieve outcomes parity with standard-risk patients. In this review, we assessed clinical trials of both doublet and triplet regimens for newly diagnosed multiple myeloma that included elderly, transplant-ineligible patients with high-risk cytogenetic abnormalities and that provided outcomes data stratified by cytogenetic risk status. We concluded that regimens containing an IMiD agent as the foundation of therapy, combined with agents that have synergistic mechanisms of action—including novel therapies—may in future investigations help overcome the poor prognosis of high-risk cytogenetic abnormalities in this vulnerable patient population.

## Introduction

Multiple myeloma (MM) is a highly heterogeneous malignancy characterized by a variable disease course [1, 2]. Specific cytogenetic abnormalities confer poor outcomes in patients with MM [3], including t(4;14), t(14;16), and del(17p), which are well validated high-risk prognostic factors [4, 5]. The International Myeloma Working Group (IMWG) molecular classification lists these abnormalities as established markers that are essential testing for all patients with MM [5]. In a retrospective study using data from the Intergroupe Francophone du Myelome database for patients aged >65 years (median, 72 years) with newly diagnosed MM (NDMM; *N* = 1890), median progression-free survival (PFS) in patients lacking both t(4;14) and del(17p) abnormalities was 24 vs 14 months for patients with t(4;14) and 11 months for patients with del(17p) (*P* < 0.001) [6]. Similarly, median overall survival (OS) in patients without either abnormality was 50 vs 32 months for patients with t(4;14) and 19 months for patients with del(17p) (*P* < 0.001).

Greater understanding of the molecular biology and development of novel therapies for MM has resulted in improved survival in recent decades [7, 8], but not all patient subgroups have benefited equally. Improvements in outcomes for patients with high-risk cytogenetic abnormalities and elderly patients have not been as great as in transplant-eligible patients who do not have high-risk cytogenetic abnormalities [3, 9]. Indeed, although overall response rates (ORRs) are often similar between patients with high-risk and standard-risk cytogenetic abnormalities [10–12], this has not always correlated to similar survival outcomes. Moreover, elderly patients with NDMM and high-risk cytogenetic abnormalities who are not eligible for transplant remain in need of treatment regimens that provide long-term benefits to PFS and OS with minimal toxicity. To date, no treatment regimen, including those containing lenalidomide or bortezomib, has demonstrated sustained and consistent survival benefits in these patients. Additionally, the relatively smaller number of elderly patients with NDMM and patients with high-risk cytogenetic abnormalities who are enrolled in prospective clinical trials has limited the quantity of available data. However, useful data can be gathered from large trials that include these patient subgroups.

The purpose of this review is to assess the available data for doublet and triplet regimens in elderly patients with transplant-ineligible high-risk NDMM and identify treatment options with the potential to overcome the poor prognosis associated with high-risk cytogenetic abnormalities.

## Methodology

To identify data regarding the use of doublet and triplet regimens in the treatment of elderly patients with transplant-ineligible NDMM and high-risk cytogenetic abnormalities, we reviewed clinical trials of regimens recommended or preferred by the National Comprehensive Cancer Network (NCCN) and European Society for Medical Oncology (ESMO) guidelines as treatment options for patients with transplant-ineligible NDMM [1, 7]. We also performed a literature search to find NDMM studies or subanalyses that included data stratified by cytogenetic risk status and patient age. Using the PubMed database, we searched for data published between 2010 and 2017 and the keywords “lenalidomide or bortezomib” and “myeloma” and “risk.” Additionally, we conducted searches for abstracts accepted by the European Hematology Association, American Society of Clinical Oncology, and American Society of Hematology congresses between 2015 and 2017, using the terms “myeloma,” “transplant,” and “cytogenetics.” After applying additional criteria—newly diagnosed, elderly, transplant ineligible, and outcomes by cytogenetic risk stratification—10 trials were identified, which together had data on treatment outcomes in approximately 650 patients with NDMM and high-risk cytogenetics (Table 1). We reported efficacy data by cytogenetic status, but reported safety data for the overall trial populations as most of the reviewed trials did not provide these data by cytogenetic status. In consideration of the narrow criteria and to avoid overlooking relevant data, clinical trials with a median patient age of <65 years (non-elderly) were included as long as patients ≥65 years of age (elderly) were also included in the trial. This age limit was chosen to define elderly in this review because 65 years of age is commonly used as a cutoff for stem cell transplant (SCT) eligibility, although it must be acknowledged that transplant may be a treatment option for fit patients older than 65 years of age as well [13–15].

**Table 1:** Select trials of doublet and triplet regimens in patients with transplant-ineligible NDMM and high-risk cytogenetic abnormalities

Trial	Patient population	Regimens	*N*	Median age (range), y	High-risk cytogenetics	Response by cytogenetics	PFS and OS by cytogenetics
Retrospective study [10]	NDMM	Rd vs RD	100	HR: 67 (48–78) SR: 63 (32–78)	HR (*n* = 16): hypodiploidy, del(13q), del(17p), t(4;14), t(14;16), or plasma cell labeling index ≥3%	ORR: HR vs SR, 81 vs 89%; *P* = 0.56 ≥ VGPR: HR vs SR, 38 vs 45%; *P* = 0.36	Median PFS: HR vs SR, 18.5 vs 36.5 mo; *P* < 0.001
Ph III E4A03 [16, 17]	NDMM	Rd vs RD	445	HR: 62 SR: 66	126 pts with FISH; HR (*n* = 21): t(4;14), t(14;16), or del(17p) deletions	ORR: HR vs SR, 75% vs 77% ≥ VGPR: HR vs SR, 30 vs 46%	Median PFS: HR vs SR, 11 vs 29 mo; *P* = 0.047 OS: HR vs SR hazard ratio, 3.48 [95% CI, 1.42–8.53]; *P* = 0.004
Ph III FIRST [18, 19]	NDMM TI	Rd continuous vs Rd18 vs MPT	1623	73 [94% ≥ 65 y]	762 pts with FISH; 19% HR [t(4;14), t(14;16), or del(17p)]	ORR: HR patients: 77% (Rd continuous) vs 67% (Rd18) vs 68% (MPT) SR patients: 81% (Rd continuous) vs 80% (Rd18) vs 71% (MPT) ≥ VGPR: HR patients: 30% (Rd continuous) vs 35% (Rd18) vs 11% (MPT) SR patients: 49% (Rd continuous) vs 47% (Rd18) vs 39% (MPT)	Median PFS: HR: Rd continuous, 8.4 mo Rd18, 17.5 mo MPT, 14.6 mo SR: Rd continuous, 31.1 mo Rd18, 21.2 mo MPT, 24.9 mo Median OS: HR: Rd continuous, 29.3 mo Rd18, 24.3 mo MPT, 35.5 mo SR: Rd continuous, 69.9 mo Rd18, 68.7 mo MPT, 53.6 mo
Ph III, SWOG S0777 [28]	NDMM without intent for immediate ASCT	RVd vs Rd	471	63 [43% ≥ 65 y]	316 pts with FISH; 33% HR [t(4;14), t(14;16), or del(17p)]^a^	NR	Median PFS: HR: RVd, 38 mo Rd, 16 mo *P* = 0.19 t(4;14): RVd, 34 mo Rd, 15 mo *P* = 0.96
Ph III VISTA [11, 12]	NDMM TI	VMP vs MP	682	71 [97% ≥ 65 y]	168 pts in VMP with cytogenetics data; 15% HR [t(4;14), t (14;16), or del(17p)]	NR	Median OS in VMP arm only: HR vs SR cytogenetics: 40.0 mo vs not reached; *P* = 0.399
Ph III Spanish GEM05MAS65 [29, 30]	Elderly NDMM	VMP vs VTP	260	HR: 72 SR: 72 [100% ≥ 65 y]	232 pts with cytogenetics data; HR (*n* = 44): t(4;14), t (14;16), and/or del(17p) SR (*n* = 188)	ORR after induction: HR, 79% SR, 82%	Median PFS: HR vs SR, 24 vs 33 mo; *P* = 0.04 Median OS: HR vs SR, 38 mo vs not reached; *P* = 0.001
Ph III GIMEMA [31]	NDMM TI	VMPT → VT vs VMP	511	71 [96% ≥ 65 y]	376 pts with cytogenetics data; 16% t(4;14), 4% t(14;16), 15% del(17p)	NR	PFS: VMP → VT vs VMP in pts with HR CAs hazard ratio, 0.98 [95% CI, 0.58–2.10]; *P* = 0.215
Ph II Spanish GEM2010 [32, 33]	Elderly NDMM	Sequential or alternating VMP and Rd	242	NR [100% ≥ 65 y]	174 pts with FISH; HR (*n* = 32): t(4;14), t (14;16), and/or del(17p) SR (*n* = 142)	ORR: Sequential (HR vs SR) 74 vs 79% Alternating (HR vs SR) 69 vs 86%	Median PFS: sequential (HR vs SR) 29.5 vs 31.5 mo; *P* = 0.9 Alternating (HR vs SR) 24 vs 33 mo; *P* = 0.03 Median OS: sequential (HR vs SR) 46 vs 63 mo; *P* = 0.1 Alternating (HR vs SR) 38.4 mo vs not reached; *P* = 0.002
Ph III [34]	NDMM TI	MPT-T vs MPR-R	668	MPT-T: 72 (33% ≥ 76 y) MPR-R: 73 (34% ≥ 76 y)	367 pts with FISH; HR (*n* = 174): del(17p), t(4;14), gain(1q21)	NR	PFS and OS: no significant difference in treatment with MPT-T vs MPR-R for all analyzed subgroups (HR CAs) Median PFS: gain(1q21) (MPT-T vs MPR-R) 17 vs 19 mo del(17p) (MPT-T vs MPR-R) 15 vs 15 mo t(4;14) (MPT-T vs MPR-R) 12 vs 14 mo Median OS: gain(1q21) (MPT-T vs MPR-R) 39 vs 50 mo del(17p) (MPT-T vs MPR-R) 41 vs 35 mo t(4;14) (MPT-T vs MPR-R) 23 mo vs not reached
Retrospective institutional study [20]	NDMM TI	CyBorD vs VMP vs Vd	122	CyBorD: 76 VMP: 73 Vd: 77	t(4;14), t(14;16), and p53 del, 21 pts (17%)	NR	Median PFS: HR vs SR, 11.8 vs 15.9 mo; *P* = 0.002 Median OS: HR vs SR, 22.4 vs 39.7 mo; *P* = 0.029

*ASCT* autologous stem cell transplant, *CA* cytogenetic abnormality, *CyBorD* cyclophosphamide, bortezomib, dexamethasone, *FISH* fluorescent in situ hybridization, *HR* high risk, *MP* melphalan, prednisone, *MPR-R* melphalan, prednisone, lenalidomide, followed by lenalidomide maintenance, *MPT* melphalan, prednisone, thalidomide, *MPT-T* melphalan, prednisone, thalidomide, followed by thalidomide maintenance, *NDMM* newly diagnosed multiple myeloma, *NR* not reported, *ORR *overall response rate, *OS *overall survival, *PFS* progression-free survival, *ph* phase, *pt* patient, *Rd* lenalidomide plus low-dose dexamethasone, *RD* lenalidomide plus high-dose dexamethasone, *Rd18* lenalidomide plus low-dose dexamethasone for 18 cycles, *RVd* lenalidomide, bortezomib, dexamethasone, *SR* standard risk, *TI* transplant ineligible, *TTP* time to progression, *Vd* bortezomib, dexamethasone, *VGPR* very good partial response, *VMP* bortezomib, melphalan, prednisone, *VMPT* bortezomib, melphalan, prednisone, thalidomide, *VT* bortezomib, thalidomide

^a^ Per preliminary analyses from available data at trial entry

## Doublet regimens: Rd and Vd

Lenalidomide–low-dose dexamethasone (Rd) and bortezomib-dexamethasone (Vd) are identified as doublet regimens for the treatment of transplant-ineligible NDMM in recent treatment guidelines [1, 7]. The NCCN guidelines recognize Rd as a preferred category 1 regimen, whereas Vd is listed in the “Useful in Certain Circumstances” category [7]. In the ESMO guidelines, Rd is listed as a “first option,” whereas Vd is not recognized [1]. The ESMO guidelines recognize melphalan-prednisone (MP) as an “other option,” but the NCCN guidelines note that melphalan-containing regimens are not standard of care in transplant-ineligible patients [1, 7]; therefore, this review did not consider MP. In NDMM trials examining doublets, efficacy outcomes in high-risk patients have typically lagged behind those patients with standard-risk cytogenetic abnormalities (Table 1). In a retrospective study of patients who received either Rd or lenalidomide–high-dose dexamethasone (RD) as initial therapy (*N* = 100), outcomes were stratified by high-risk vs standard-risk cytogenetic abnormalities, with high-risk cytogenetic abnormalities defined as presence of del(17p), t(4;14), t(14;16), hypodiploidy, plasma cell labeling index ≥3%, or monoallelic loss of chromosome 13 or its long arm [10]. Fifty patients (50%) proceeded to SCT after induction. ORR was similar in patients with high-risk cytogenetic abnormalities (*n* = 16; median age 67 years) and those with standard-risk cytogenetic abnormalities (*n* = 84; median age 63 years); 81 vs 89%, respectively (*P* = 0.56). However, PFS was significantly longer in the standard-risk group vs the high-risk group (36.5 vs 18.5 months, respectively; *P* < 0.001). In an analysis that censored data from patients who underwent SCT, the PFS findings were similar to those in the overall analysis: 36.9 months for standard-risk patients vs 18.5 months for high-risk patients (*P* = 0.002). No safety data were reported in this publication.

The open-label, phase 3 E4A03 study (*N* = 445) compared RD with Rd in patients with NDMM [16]. The median age of patients in the RD group was 66 years (53% ≥65 years) and was 65 years (51% ≥65 years) in the Rd group. Eligible patients could receive SCT after the first four cycles of therapy; those who underwent SCT (*n* = 167; 38%) discontinued the study per protocol. After a median follow-up of 12.5 months, Rd was superior to RD for OS (*P* = 0.0002) and was associated with less toxicity. Jacobus et al. performed a subanalysis on the clinical significance of cytogenetic risk status in this study population [17]. Fluorescent in situ hybridization (FISH) analysis data were available for 126 patients, with 21 classified as high risk based on the presence of t(4;14), t(14;16), or del(17p). ORR was similar in the two cytogenetic abnormality risk groups (75% for standard risk and 77% for high risk), whereas the standard-risk group had a longer median PFS (29 vs 11 months; *P* = 0.047) and a higher 2-year OS rate (91 vs 76%) vs the high-risk group. Overall, toxicities were reported more frequently with RD than Rd. Significantly more patients in the RD group had grade ≥3 nonhematologic toxicities than patients in the Rd group (65 vs 48%; *P* = 0.0002), including deep vein thrombosis or pulmonary embolism (26 vs 12%; *P* = 0.0003) and hyperglycemia (11 vs 6%; *P* = 0.09). Rates of neuropathy were similar between the treatment groups (2 vs 2%; *P* = 0.1). More patients in the RD group (27%) discontinued treatment due to adverse events (AEs) than patients in the Rd group (19%) [16].

The phase 3 FIRST trial compared Rd continuous vs Rd for 18 cycles (Rd18) vs MP-thalidomide (MPT) in transplant-ineligible patients with NDMM (*N* = 1623) [18]. Across all treatment arms, the median age was 73 years. In the Rd continuous and Rd18 groups, 94% of patients were aged ≥65 years, and in the MPT group, 95% of patients were aged ≥65 years. For patients with high-risk cytogenetic abnormalities, Rd continuous resulted in a numerically higher ORR than both MPT and Rd18 (77 vs 68 vs 67%, respectively), but did not demonstrate a significant improvement in PFS or OS vs MPT [19]. In patients with standard-risk cytogenetics, ORRs were 81, 71, and 80%, respectively. Despite the similar ORRs observed in patients with high-risk and standard-risk cytogenetics, PFS was worse in high-risk patients. The 4-year PFS rates were 34.7, 11.8, and 15.3%, respectively, for patients with standard-risk cytogenetics, compared with 3.0, 10.0, and 0%, respectively, for patients with high-risk cytogenetic abnormalities. These data further indicate that the Rd doublet alone is unable to overcome the poor PFS prognosis associated with high-risk cytogenetic abnormalities. As reported in the initial publication, MPT was associated with higher rates of hematologic AEs than Rd continuous and Rd18 [18]. There was a higher frequency of grade 3/4 neutropenia reported with MPT (45%) than with either Rd continuous (30%) or Rd18 (26%). In the Rd continuous group, 32% patients had grade 3/4 infections vs 22% with either Rd continuous or 17% with MPT [19].

Although limited data exist for Vd in randomized studies, Vd has been examined in elderly patients with transplant-ineligible NDMM. Jimenez-Zepeda et al. conducted an institutional study examining the use of cyclophosphamide-bortezomib-dexamethasone (CyBorD), bortezomib-MP (VMP), and Vd in transplant-ineligible NDMM (*N* = 122) [20]. The median age at diagnosis for patients in the study was 76, 73, and 77 years, respectively. High-risk cytogenetic abnormalities, defined as t(4;14), t(14;16), or p53 del, were identified in 21 patients overall (17%). Compared with standard-risk patients, high-risk patients had significantly shorter median PFS (11.8 vs 15.9 months; *P* = 0.002) and median OS (22.4 vs 39.7 months; *P* = 0.029). The most common AE across all cohorts was peripheral neuropathy (42.8% in the CyBorD group, 66% in the VMP group, and 55% in the VD group; *P* = 0.03).

In the reviewed studies, median PFS for high-risk patients treated with doublets ranged from 8 to 19 months, compared with a range of 21 to 37 months in standard-risk patients. It must be noted that, in both the retrospective study by Kapoor et al. and the E4A03 study, substantial proportions of patients proceeded to SCT. Nevertheless, these data demonstrate that current doublet regimens are suboptimal, because neither Rd nor Vd alone is enough to overcome the poor prognosis associated with high-risk cytogenetic abnormalities in patients with NDMM, most of whom were elderly and not eligible for transplant. However, triplet regimens containing novel agents, as discussed below, may better improve survival outcomes in this patient population.

## Triplet regimens

Elderly patients with MM are a heterogenous population, and they range from fit to frail [21, 22]. Frail elderly patients may not be able to tolerate triplet regimens as well as those who are not frail may be able to. With that noted, the NCCN MM guidelines recommend triplet regimens over doublet regimens in patients who can tolerate them [7]. An IMWG consensus statement on the treatment of MM with high-risk cytogenetic abnormalities recommends a triplet regimen that includes a proteasome inhibitor, an IMiD agent, and dexamethasone for the treatment of patients with NDMM and high-risk cytogenetic abnormalities [4]. Despite the limited efficacy of Rd or Vd alone in patients with high-risk cytogenetic abnormalities, promising results have been demonstrated in patients with relapsed or refractory MM and high-risk cytogenetic abnormalities using Rd or Vd as a backbone regimen [23–27].

The open-label, multicenter, phase 3 SWOG S0777 trial compared Rd with RVd in patients with NDMM without intent for immediate autologous SCT (ASCT) [28]. The median age of patients in both treatment groups was 63 years; 43% of the patients evaluable for efficacy were ≥65 years of age. Median follow-up was 55 months. Patients treated with RVd had significantly longer median PFS than those treated with Rd (43 vs 30 months; *P* = 0.0018). Data from FISH analyses conducted at trial entry were available for 316 patients; 33% had ≥1 high-risk cytogenetic abnormality—either t(4;14), t(14;16), or del(17p). Among evaluable high-risk patients (*n* = 44), there was numerical superiority in the RVd group compared with the Rd group for median PFS (38 vs 16 months), but this was not significant (*P* = 0.19). A similar treatment difference in median PFS was seen in patients with t(4;14) specifically (*n* = 17): 34 months in the RVd group vs 15 months in the Rd group (*P* = 0.96). Overall, 75% of patients in the Rd group experienced grade ≥3 AEs vs 82% in the RVd group. Patients treated with RVd had a greater frequency of grade ≥3 neurologic toxicity than patients treated with Rd (33 vs 11%; *P* < 0.0001).

The phase 3 VISTA trial compared the efficacy of VMP with that of MP for the treatment of NDMM (*N* = 682); the study population was elderly, with a median age of 71 years and 97% of patients ≥65 years [12]. Of patients with FISH data who were treated with VMP (*n* = 168), 15% were considered high risk due to presence of t(4;14), t(14;16), or del(17p). In patients treated with VMP, OS trended longer among the standard-risk patients than the high-risk patients (median OS, not reached vs 40.0 months; hazard ratio (HR), 1.346 [95% confidence interval (CI), 0.674–2.687]; *P* = 0.399) [11]. As reported in the initial publication, there was no statistical difference in time to progression between patients treated with VMP who had high-risk cytogenetic abnormalities and those who had standard-risk cytogenetics (median time to progression, 19.8 vs 23.1 months; HR, 1.297 [95% CI, 0.55–3.06]; *P* = 0.55) [12]. In the VMP group, 91% of patients had a grade ≥3 treatment-emergent AE vs 80% in the MP group [11]. Neutropenia was the most common grade 3/4 AE in either treatment arm, and was reported in 12% of patients receiving VMP and 11% of patients receiving MP.

In the phase 3 Spanish GEM05MAS65 trial, patients aged ≥65 years with NDMM were treated with either VMP or bortezomib-thalidomide-prednisone (VTP) as induction regimens (*N* = 260) [29]; patients were then randomized to maintenance with bortezomib-prednisone or bortezomib-thalidomide (VT). FISH analysis data were available for 232 patients [30]; 19% had high-risk cytogenetic abnormalities (*n* = 44), defined as t(4;14), t(14;16), or del(17p). The median age of both standard- and high-risk patients was 72 years. ORR after induction was similar between the standard-risk and high-risk groups (82 vs 79%), regardless of treatment. The high-risk group had a significantly shorter median PFS from first randomization (24 vs 33 months; *P* = 0.04) and median OS (38 months vs not reached; *P* = 0.001) compared with standard-risk patients. Neutropenia was reported more frequently with VMP than VTP (39 vs 22%; *P* = 0.008), as was thrombocytopenia (27 vs 12%; *P* = 0.0001) [29]. As for nonhematologic toxicities, VMP was associated with a higher incidence of infections than VTP (7 vs 1%; *P* = 0.01) but a lower incidence of cardiac events (0 vs 8%; *P* = 0.001). During the maintenance phase, there were no grade ≥3 hematologic AEs.

The phase 3 GIMEMA trial compared the efficacy of bortezomib-MPT (VMPT) followed by VT maintenance with that of VMP in patients with transplant-ineligible NDMM (*N* = 511) [31]. The median age of the study population was 71 years, with 96% aged ≥65 years. FISH data were available for 376 patients; 15% had del(17p), 16% had t(4;14), and 4% had t(14;16). There was no significant PFS benefit with VMPT followed by VT vs VMP among high-risk patients (HR, 0.98 [95% CI, 0.58–2.10]) or standard-risk patients (HR, 0.69 [95% CI, 0.46–1.02]), suggesting that adding a fourth agent to induction therapy may not provide added benefit over a triplet regimen. The frequency of grade 3/4 hematologic AEs was similar between the VMPT followed by VT and VMP groups (47 vs 41%; *P* = 0.20). However, VMPT followed by VT was associated with more frequent grade 3/4 nonhematologic AEs (46 vs 33%; *P* = 0.003); grade 3/4 cardiologic events were reported in 10% of patients in the VMPT followed by VT group vs 5% in the VMP group (*P* = 0.04).

The phase 2 Spanish study GEM2010 (*N* = 242) compared sequential vs alternating VMP with Rd in patients with NDMM [32]. There were 233 patients who were evaluable for safety and efficacy, and all were aged ≥65 years. FISH data were available for 174 patients [33]. Of these patients, 32 had high-risk cytogenetic abnormalities, whereas 142 had standard-risk cytogenetic abnormalities. Between high-risk and standard-risk patients, ORRs did not differ significantly. High-risk and standard-risk patients had similar ORRs in both the alternating treatment arms (69 and 86%, respectively) and the sequential treatment arms (74 and 79%). In the alternating treatment arm, patients with high-risk cytogenetic abnormalities had a shorter median PFS than those with standard-risk cytogenetic abnormalities (24 vs 33 months; *P* = 0.03); patients with high-risk cytogenetic abnormalities also had shorter median OS than those with standard-risk cytogenetic abnormalities (38.4 months vs not reached; *P* = 0.002). In the sequential treatment arm, however, there was no significant difference between standard-risk and high-risk patients in their median PFS (31.5 vs 29.5 months, respectively; *P* = 0.9) or median OS (63 vs 46 months, respectively; *P* = 0.1). Safety data were reported by age group [32]. More patients >80 years of age (63%) discontinued the trial due to toxicity or informed consent withdrawal than patients aged 65–75 (30%) or those aged 75–80 years (30%).

A multicenter, randomized phase 3 trial by Zweegman et al. examined MPT followed by thalidomide maintenance (MPT-T) vs MP-lenalidomide followed by lenalidomide maintenance (MPR-R) in patients with transplant-ineligible NDMM (*N* = 668) [34]. In the MPT-T arm (*n* = 318), the median age of patients was 72 years (33% aged ≥76 years), and in the MPR-R arm (*n* = 319), median age was 73 years (34% aged ≥76 years). FISH was performed in 73% of patients in the MPT-T arm and 78% in the MPR-R arm, with presence of del(17p), t(4;14), or gain(1q21) being classified as high risk. The MPT-T arm had 87 standard-risk patients and 88 high-risk patients, whereas the MPR-R arm had 106 standard-risk patients and 86 high-risk patients. In subanalyses of MPT-T vs MPR-R for both OS and PFS based on age and cytogenetic risk status, no statistical difference in outcomes between the treatment groups was found across any of the investigated subgroups. In the overall population, there was no statistical difference in PFS with MPT-T (20 months) vs MPR-R (23 months). Outcomes data for patients with high-risk cytogenetic abnormalities were provided for each cytogenetic abnormality individually, with each abnormality negatively impacting PFS (*P* < 0.01). Among patients in the MPT-T arm, PFS was 17, 15, and 12 months for gain(1q21), del(17p), and t(4;14), respectively, and 19, 15, and 14 months for patients treated with MPR-R. Findings from the FIRST trial support these data and show that MPT cannot overcome the adverse PFS prognosis of high-risk cytogenetic abnormalities. The rate of grade 3/4 AEs was 81% in the MPT-T group vs 86% in the MPR-R group (*P* = 0.13). Grade 3/4 hematologic AEs were more frequently reported with MPR-R vs MPR-T, including neutropenia (64 vs 27%; *P* < 0.001), thrombocytopenia (30 vs 8%; *P* < 0.001), and anemia (14 vs 5%; *P* < 0.001).

In the reviewed trials that examined triplet regimens, median PFS for patients with high-risk cytogenetic abnormalities ranged from 12 to 38 months. Among the studies that included data for standard-risk patients, median PFS ranged from 32 to 33 months. Although these are limited data, the reported median PFS range for high-risk patients reached a longer period (38 months) than that noted from the doublet trials (19 months). Notably, the longest reported median PFS (38 months) in patients with high-risk cytogenetic abnormalities was achieved with RVd, as reported in the SWOG S0777 trial, which was the only reviewed trial that included the recommended triplet regimen by the IMWG (a proteasome inhibitor, IMiD agent, and dexamethasone). This further suggests that elderly patients with high-risk cytogenetics may benefit from regimens that combine novel agents.

## Discussion and future strategies

Over half of patients with myeloma are considered elderly (≥65 years), with a median age at diagnosis of 69 years, and approximately 15–20% of patients newly diagnosed with myeloma have high-risk disease, including the presence high-risk cytogenetic abnormalities among other prognostic features [35, 36]. Advanced age may preclude many of these patients from receiving SCT, which is commonly reserved for patients <65 years of age [13–15]. These patients are routinely seen in clinical practice, but they are underrepresented in clinical trials, making treatment decisions difficult. Elderly patients with NDMM and high-risk cytogenetic abnormalities need effective and tolerable treatment regimens to help them overcome the poor prognosis imparted by their high-risk cytogenetic status, but there are many challenges in the treatment of these patients (Table 2). Additionally, the fragmented data from a limited number of trials demonstrate that no therapeutic modality in NDMM has consistently shown improvements in PFS for elderly patients with high-risk cytogenetic abnormalities, and these patients have not achieved survival outcomes parity with standard-risk patients in clinical trials. Lenalidomide and bortezomib have emerged as cornerstones of MM care, but the above review of the limited data available with doublet regimens demonstrate that neither Rd nor Vd alone is enough to mitigate the poor prognosis of high-risk cytogenetic abnormalities. However, because some novel agents have great tolerability and synergistic mechanisms of action (MOAs), patients may benefit from regimens that use a combination of these novel agents [19, 37]. Indeed, a 2016 consensus statement by the IMWG recommends that patients with NDMM and high-risk cytogenetic abnormalities receive a triplet regimen containing a proteasome inhibitor, dexamethasone, and lenalidomide or pomalidomide [4]. Going forward, it will be important to further explore the efficacy of such triplets and identify the optimal regimens for these patients.

**Table 2:** Key challenges in the treatment of patients with multiple myeloma with high-risk cytogenetic abnormalities

Challenge	Explanation
Inconsistent definitions for high-risk CAs	The lack of consensus on precisely which CAs are considered high risk leads to variable inclusion of CAs in clinical studies, complicating data interpretation by clinicians
Limited data	Past clinical trials have not consistently included patients with high-risk CAs. In studies that do include these patients, a full subanalysis may not be executed, and the small number of patients with high-risk CAs makes it difficult to compare outcomes with SR patients or overall study populations
High cost of testing for CAs	Standard bone marrow examination, required for FISH analysis, has become more expensive [39]
Heterogeneity of CAs	Multiple CAs impart poor prognosis. Treatments may help overcome an aspect of the poor prognosis imparted by one CA but not others, or may help in TE patients but not TI patients; this requires careful consideration of therapy
Lack of treatment guidelines	Although the NCCN and ESMO MM guidelines both recognize cytogenetic abnormalities as prognostic factors, neither provides categorized treatment recommendations for patients with TI NDMM and high-risk CAs [1, 7]

*CA* cytogenetic abnormality, *ESMO* European Society for Medical Oncology, *FISH* fluorescent in situ hybridization, *MM* multiple myeloma, *NCCN* National Comprehensive Cancer Network, *NDMM* newly diagnosed multiple myeloma, *SR* standard risk, *TE* transplant eligible, *TI* transplant ineligible

Multiple factors make interpretation of data from high-risk patients difficult. Risk stratification in MM remains fluid, and the lack of consistency in the methods used to stratify patients into cytogenetic risk categories confounds interpretation of the data. The IMWG has identified multiple criteria used by different methods to assess risk status involving various factors [4]. The existence of multiple methods and the inconsistency of clinical study adherence to them has resulted in a potential for variability in results and data interpretation. Furthermore, cytogenetic abnormalities are often not considered as inclusion criteria in most clinical trial protocols and outcomes by cytogenetic risk are often exploratory in nature. Discrepancies among risk stratification guidelines are limiting; there is no direct way to compare patients across risk groups with the different methods, and not all high-risk groups impart the same prognosis. Additionally, cytogenetic data analyses are often not performed at one centralized facility, even among patients within the same study. Further, there is a lack of prospective trial data that inform the use of available methodologies to comprehensively profile patients and select regimens that will provide the most benefit.

Another challenge in the interpretation of data for patients with transplant-ineligible high-risk NDMM is identification of the optimal efficacy endpoint. High-risk and standard-risk patients have similar response rates to treatments, but despite this, there is still disparity in their survival outcomes. Clinical trials commonly rely on PFS and OS, but other endpoints may be valuable. Chakraborty et al. have determined that depth of response and minimal residual disease are relevant endpoints for certain high-risk patients [38]. Additionally, data from high-risk patients and elderly patients are often reported for the entire study population rather than for each individual treatment arm. Although this facilitates identification of overall trends, such as worse outcomes overall for high-risk patients, it makes it difficult to ascertain the effect of treatment. The small number of patients with high-risk cytogenetic abnormalities included in clinical trials presents another challenge: making statistically significant conclusions with small populations is more difficult than it is with larger standard-risk or intention-to-treat populations. Data from trials examining triplet regimens containing the Rd or Vd backbone with newer agents (carfilzomib, elotuzumab, daratumumab, and ixazomib) for the treatment of relapsed or refractory MM have shown promising results in patients with high-risk cytogenetic abnormalities, and we eagerly anticipate the results of such combinations in NDMM studies [23–27]. The available data suggest that using an IMiD agent as the foundation of combination therapy with drugs that have synergistic MOAs may overcome the poor outcomes imparted by high-risk cytogenetic abnormalities.

## Conclusion

No treatment regimen for transplant-ineligible NDMM has been consistently shown to improve outcomes in patients with high-risk cytogenetic abnormalities. Incorporating promising emerging agents—such as monoclonal antibodies, checkpoint inhibitors, and vaccines [37]—in combination regimens with synergistic MOAs may benefit patients with transplant-ineligible NDMM and high-risk cytogenetic abnormalities in future investigations. However, to better identify optimal regimens for these patients, further consensus is needed to consolidate and refine risk assessment guidelines, which should improve analytical and design uniformity among clinical trials. It must also be considered that the age cutoff in this review of ≥65 years to define elderly patients was based on the standard convention that SCT is generally reserved for patients aged <65 years. In practice, treatment decisions for patients with high-risk cytogenetic abnormalities should be based on clinical assessment of each patient’s frailty; combination regimens including the previously mentioned emerging agents are not likely to be tolerable for frail patients but may be good options for fit elderly patients. Per the recommendations of the IMWG consensus statement, further NDMM clinical trials, especially those evaluating novel agents, should continue to enroll these patients and either conduct appropriate post hoc risk-stratified outcomes analyses or directly apply risk-stratified treatment [5]. Finally, data for elderly patients and high-risk patients should be reported not only in the context of the overall study population but also for each treatment arm.
